# The COVID-19 Context Calls for a Broader Range of Healthcare Chaplaincy Models: An Exploratory Translational Study Utilizing Evolutionary Psychology and Social Neuroscience Loneliness Research

**DOI:** 10.1177/1542305020962417

**Published:** 2020-11-23

**Authors:** Ann K. Riggs

**Affiliations:** Howard County General Hospital, USA

**Keywords:** COVID-19, loneliness, healthcare chaplaincy, evolutionary psychology, social neuroscience

## Abstract

Shifts in chaplain requests from patients and families and lack of engagement by staff in now traditional support forms in the COVID-19 context suggest that new insights and resourcing are needed. This exploratory translational study suggests that the evolutionary psychology of R. I. M. Dunbar and the social neuroscience of J. T. Cacioppo, his collaborators, and successors and their concerns for human loneliness have potential for use in development of effective healthcare chaplaincy practice in the COVID-19 context.

## Initial Observations

As the COVID-19 pandemic was just beginning, a shift in chaplain requests from patients and families began at our community hospital. There seemed to be fewer occasions focused on help in the management, working through, and letting go of waves of intense feelings of anxiety, fear, frustration, or grief, sometimes seen as the classic work of hospital chaplains. (Bard, 2020) There were more requests for brief, more formal prayer/liturgies, or, quite differently, for lighter exchanges with a chaplain. Lockdown phone visits of both types have been responded to with surprising warmth and gratitude. At times, patients have begun our phone visit by eagerly thanking me simply for calling in response to their request entered in the admission process. At others, calling a patient in response to a referral from the patient’s nurse, I hear the patient’s voice transform from downhearted or oppositional to a more normal speech tone and pace in response to light banter and joking that produce laughter. I sometimes arrange with a patient that I will call them again in a few hours, once more with no expectation of a strong emotional processing and release.

Although patients and family members are of diverse Christian commitments, during this COVID-19 context, offering ministry over the telephone,I have repeatedly made successful use of selections of short prayers, around 100 words or fewer, from formal liturgies for ministry to the sick and at a death, from collections containing traditional personal devotional prayers, and using similar formal language and cadence in impromptu prayer. These prayers often pair with a brief biblical selection such as Psalm 24:1–5, in the King James Version or other poetic formulation. Depending on the needs assessed I may or may not include a prayer focused on distinctive characteristics and needs of the patient and family, but I always seek to verbally locate the patient and family in a broader group–a community.

One patient visit included both light social interchange and more formal prayer elements. The patient, “Lucia”, was recovering from COVID-19 and still unable to receive visitors: family, friends, local faith-related volunteers, or employee chaplains. She was Spanish speaking, so our phone visit included an interpreter. The patient was mid-fifties and Catholic. She had requested a chaplain visit but had not named any specific concern or need. Almost as soon we began exchanging initial introductions and I asked how Lucia was doing today, her voice became lighter in tone and timbre. She started laughing. We turned to prayer as she requested, the *Memorare*,^1^ which Lucia knew in Spanish. Part way through our prayer together Lucia was repeating in English close to the form I had used after the interpreter translated each clause. We concluded our visit soon afterwards with mutual good wishes for the rest of the day and generous thanks and blessings for me from Lucia.

As the pandemic has progressed staffers have seemed strained and disoriented. All are urged to avoid taking breaks with others and refrain from congregating; stand 6 feet away from patients and colleagues whenever possible; wear a face mask when within 10 feet of others. Some frontline staff with vulnerable loved ones at home stay in donated hotel rooms and recreational vehicles (RVs) to physically distance from family and friends.An Emergency Department (ED) physician in our community hospital articulated feeling disconnected from patients and the needs that had brought them in, due to the extensive personal protective equipment (PPE) the COVID-19 context required everyone to wear: one or more gowns, N95 respirator and eye shield or powered air-purifying respirator (PAPR), double or triple layers of gloves. 

Johns Hopkins psychologist George S. Everly summarizes:

Every disaster brings psychological casualties that far outnumber physical ones. Common reactions include depression, grief, guilt, generalized anxiety and post-traumatic stress. With regard to this pandemic, we’re seeing all of these things. If that weren’t enough, many people have lost their jobs, and they may have preexisting psychological problems. There could be an uptick in physical, emotional and sexual abuse, causing more angst. (2020)

Yet increased opportunities to process feelings with chaplains, or with social workers, do not seem to be as accepted or utilized as has been hoped.

What does it mean? Are there now more patients and family members who are instrumental grievers and fewer intuitive grievers than previously? (Doka & Martin, 2010) That is, are there more people aided by processing loss and grief or other strong emotions through ritual, arts, or other mediatory processes rather than directly by emotional articulation and manifestations such as tears shared with an attentive listener? Are staffers trying to appear stronger or less in need of help managing, working through, and letting go their feelings than they really are? Are they embarrassed to seek support?

In his editorial for the June 2020 issue of the *Journal of Pastoral Care &Counseling*, “COVID-19 and a new normal,” Terry Bard notes that something is different in the COVID-19 landscape of hospital healthcare chaplaincy. Bard assesses that chaplains are now wearing two hats. The first hat is providing support of persons in their management, working through, and letting go of troubling emotions. Bard identifies another form of needs that does not seem to respond to this familiar style of care. Bard describes this as moral distress, and potentially post-traumatic stress disorder (PTSD), associated with dismay at the inequities and inadequacies of the global COVID-19 medical preparation and response (Bard, 2020; *COVID-19: Global reflections on faith, health, and justice*, 2020).

This study likewise engages observation that the COVID-19 healthcare chaplaincy care context makes different demands on healthcare chaplains. Drawing on the evolutionary psychology of R. I. M. Dunbar (Bzdok & Dunbar, 2020; Dunbar, 2020; Murthy, 2020) and the social neuroscience of J. T. Cacioppo and his collaborators and successors (J. Cacioppo & Patrick, 2008; S. Cacioppo et al., 2015), this exploratory translational study turns inquiry in a different direction. I suggest insights and possible applications for use of evolutionary psychology and social neuroscience in healthcare chaplaincy in the COVID-19 context.

Rubio et al. (2011) frame a broad definition of translational research in the American Academy of Medical College’s journal *Academic Medicine*:

Translational research fosters the multidirectional integration of basic research, patient-oriented research, and population-based research, with the long-term aim of improving the health of the public. T1 research expedites the movement between basic research and patient-oriented research that leads to new or improved scientific understanding or standards of care. T2 research facilitates the movement between patient-oriented research and population-based research that leads to better patient outcomes, the implementation of best practices, and improved health status in communities. T3 research promotes interaction between laboratory-based research and population-based research to stimulate a robust scientific understanding of human health and disease. (p. 471).

Dunbar’s evolutionary psychology and the field of social neuroscience founded by J. Cacioppo, and continued after his death, report and synthesize extensive observational and laboratory basic science research. In the recently published *Together: The healing power of human connection in a sometimes lonely world*, Vivek Murthy, MD, a former US surgeon general, offers the research of Dunbar, J. Cacioppo, and other basic science in a translational form for popular reading and addressed to stimulate improved population health (T2) (Murthy, 2020). My exploratory translational study (T1) suggests that the evolutionary psychology of Dunbar and the neuroscience of J. Cacioppo, his collaborators, and successors, have protentional for use in development of healthcare chaplaincy practice in the COVID-19 context.

## Evidence-based Loneliness Theories from Evolutionary Psychology and Social Neuroscience Basic Science of Loneliness

Dunbar is an academic scientist, publishing hundreds of research articles in anthropology and psychology, and a popular science writer. Dunbar’s research is “basic science” from the point of view of healthcare sciences. Dunbar locates the development of contemporary human sociality in the context of the sociality, the culture, of our evolutionary ancestors.

Recently, Bzdok and Dunbar reviewed the neurobiology of social distance in relation to the public health demands and personal care needs in the COVID-19 context. Dozens of studies have documented that mortality and morbidity are impacted by our social connectivity. Blood pressure, immune system status, and physical resilience in response to surgery or illness are demonstrably impacted by sociality (Bzdok & Dunbar, 2020, pp. 1–4)

In other primates, mutual grooming of one another triggers endorphins, hormones that reduce pain and extend pleasure, creating a feeling of well-being in both the giver and the receiver of the grooming. Humans may support their sociality by grooming one another, as well.

In Chicago’s North Lawndale neighborhood, Carter’s Barbershop is the venue for the Barber Shop Show, a weekly radio show and podcast. The conviviality of the barber shop makes it a successful locale for “a weekly dose of real talk, straight from the shop floor. No punches are pulled and no topic is considered off-topic,” such as one on raising black boys in the racially distorted context of the United States (Steele, 2016). COVID-19 has made these culturally well-established venues for mutuality dangerous and inaccessible.

Humans have many other means for endorphin-producing, mutual connection. Dancing and singing in secular or religious contexts and common prayer or liturgy are examples for such connection that have become problematic in the COVID-19 context. But laughing, emotional storytelling, and using spoken language in other ways to create social bonding are not inaccessible in the current moment. Some research suggests behavioral synchrony, as when all move, sit, kneel, or stand together, amplifies the level of endorphin release (Bzdok & Dunbar, 2020). As areas open incrementally after the most intense COVID-19 lockdowns periods, and before most religious institutions that use behavioral synchrony in public worship could return to indoor congregational assembly, the use of the behavioral synchrony of kneeling or standing in memory of George Floyd by the White Coats for Black Lives movement is notable (Novick, 2020).

The characteristic focus on mutuality in Dunbar’s research is of interest for the context of healthcare chaplaincy. Dunbar’s research and discussion are not of one-way interactions but of relationships where each gives and each receives. Although these elements may not be symmetrical, each party is contributor as well as beneficiary. These are friendships and communities rather than transactional interactions.

Dunbar and others have noted that humans ordinarily maintain personal relationships at varying distances from themselves: intimate, relational, collective. A small number of intimate relationships are with closest family members and friends. A higher number of relationships, perhaps around 50, are with a wider relational group of colleagues, community members, and less close friends and family. The widest group may be spoken of as collective or as expressing identity: religion, profession, sports team supported, tribe. Disruptions in our intimate, relational, collective relationships can lead not to solitude, a positive, comfortable state of separation, but intimate, relational, and/or collective loneliness (J. Cacioppo & Patrick, 2008).

In a scientific context, loneliness may be defined as a sensed “discrepancy between an individual’s preferred and actual social relations (Peplau & Perlman, 1982). This discrepancy then leads to the negative experience of feeling alone and/or the distress and dysphoria of feeling socially isolated” (S. Cacioppo et al., 2015, pp. 238–239).Loneliness is known to increase risk of depression, addictions, stroke, cognitive decline, and other physical and psychological disorders (Bzdok & Dunbar, 2020). This earlier research suggests we expect the physical isolation demanded by the COVID-19 context can readily conduce to intimate, relational, and/or communal loneliness on the part of patients, families, staff, and the wider community.

Loneliness can be self-amplifying. Loneliness can lead to distortion in perception of the intentions or meanings of others’ actions. In *Loneliness: Human nature and the need for social connection*, J. Cacioppo and W. Patrick (2008) make the observational and clinical research of J. Cacioppo and others translationally available to the popular reader (T2).

Neurologically, feeling lonely increases a person's attentiveness to social cues, as being hungry increases attentiveness to food. But loneliness also decreases a person’s ability to read social clues accurately. People feeling lonely have been documented to be less accurate in reading facial expressions and in assessing meaning in voice timbre. When feeling lonely we are more attuned to the negative, unpleasant, and threatening but less accurate in recognizing it correctly, potentially misreading positive cues negatively, leading to a downward spiral of loneliness. Further, loneliness can also lead us to negatively biased assessments of our own performance or capacity. We become more fragile and self-critical (J. Cacioppo & Patrick, 2008).

## Documented Loneliness Care in Other Disciplines

While COVID-19 is new, and we are all trying to figure out ways forward, some T1 research translating basic science on loneliness into loneliness care has been carried out in other settings and can be assessed for possible usefulness to healthcare chaplaincy in the COVID-19 context. S. Cacioppo et al. (2015) reviewed documented interventions to reduce chronic loneliness between 1970 and 2009.

Sorting through the various documented studies, they identified 20 that met rigorous criteria for randomized comparison design. Interventions were each in one of four primary types of interventions thought to reduce loneliness: (a) those that increase opportunities for social contact; (b) those that enhance social support; (c) those that focus on improving social and communication skills; (d) those that address maladaptive social cognition (cognitive behavioral therapy). Of the interventions to reduce loneliness studied, interventions designed to address maladaptive social cognition were associated with the largest effect size (mean effect size = −.598), almost four times as effective as social support interventions to reduce loneliness (mean effect size = −.162), the next most effective form. Neither interventions to reduce loneliness through increased opportunities for social contact nor interventions to reduce loneliness by teaching improved social and communication skills showed statistically significant effectiveness in these studies (S. Cacioppo et al., 2015).

Cognitive behavioral therapy (CBT) involves strategies to change thinking patterns. Efforts might include:

Learning to recognize distortions in one’s thinking that are creating problems, and to reevaluate assessments in light of further information.

Gaining better insight into the behavior and motivation of others.

Using enhanced problem-solving skills in demanding situations.

Developing greater confidence is one's own abilities. (American Psychological Association (APA), 2017; Young, 1982)

S. Cacioppo et al. (2015) note three studies of cognitive behavioral therapy interventions that might inform translation of evolutionary psychology and social neuroscience in healthcare chaplaincy in the COVID-19 context.

In the 2000s, research was carried out on cost-effective, interventional programming to support the successful completion of US Navy initial training (boot camp) by young recruits (Williams et al., 2004; Williams et al., 2007). Some recruits had been identified by trainers during the first days of boot camp as at risk for failure to complete training and pass all required skill and performance assessments successfully. Two randomized studies, the first on small groups of control, at-risk interventional and at-risk non-interventional Navy recruits, and the second similar but carried out with larger groups of study subjects were carried out.

These studies made use of established instruments. The first study used Beck Depression Inventory–2nd Edition, Perceived Stress Scale, Revised UCLA Loneliness Scale, Sense of Belonging Inventory-Psychological, Coping Style Questionnaire, and Attachment Style Questionnaire, plus purpose-created weekly report forms in which participants documented their own sense of their current emotional reactivity and stress severity. The interventions provided CBT designed for the context and subjects, young men and women hoping to qualify for full acceptance into the US Navy.

Using a brief, accessibly written manual, homework assignments, and 45 minutes of group instruction each week for 8 weeks, control, at-risk intervention, and at-risk non-intervention groups of recruits were introduced to, discussed, and practiced cognitive substitutions and advice. Substitution of an unrealistic negative thought, such as “I am a total failure,” with a realistic positive thought, such as “I’m often successful at the things I do,” was advocated. Homework assignments included directions such as “Each week, get to know better one of your shipmates by talking to the shipmate, not criticizing the shipmate, showing interest and listening.” To avoid positive results tied only to enhanced small-group participation, non-intervention participants and the control group in the study were active in 45 minutes of other small-group activities.

The Navy intervention produced significant improvement in the overall performance of the recruits in their training. In their first study, at-risk recruits who participated in the intervention performed at the same performance success as the control group, around 86% and 84% success rates respectively. The at-risk recruits in the non-intervention group scored only a 74% pass rate. The change in the “sense of belonging” and “loneliness” scores were among the strongest changes (Williams et al., 2004).

The Navy intervention was made in a specialized setting and with primarily young adult participants. Yet in some ways the Navy basic training context is closely comparable to the COVID-19 context in general and the COVID-19 healthcare context in specific. The isolation participants experience is externally imposed, disrupting established and perhaps formerly highly successful socialization patterns. Loneliness-creating isolation from personal contacts is combined with on-going need for teamwork. Although the Navy trainees are only practicing, both COVID-19 healthcare and military contexts are high-stress, life-and-death environments. The intervention with Navy recruits appears pertinent to the COVID-19 healthcare context.

The third CBT study S. Cacioppo et al. identify as significantly successful in documenting loneliness reduction is also a small-group study, carried out in a nursing home in Taipei, Taiwan. Chiang et al. (2009) studied the effects of reminiscence therapy on psychological well-being, depression, and loneliness among elderly institutionalized males.

As commonly performed in elder care settings, reminiscence therapy may utilize multiple senses, sight, sound, touch, taste, or smell. Individuals recall memories from earlier in their lives and may be asked to share these with a group of current neighbors. The therapy may be carried out through conversational formats or more structured modes (Elder Care Alliance, 2017).

The report on the reminiscence study of CBT positively impacting loneliness carried out in Taipei was similar to the Navy recruit study in that it utilized eight small-group sessions. Volunteer participants shared stories from their past and were encouraged to display affirmations toward other participants. As the group members were all illiterate, there were no written materials in the program. All members were aged 65 or older and all were male (Chiang et al., 2009).

The loneliness measure for the study was the Revised University of California Los Angeles loneliness scale (RULS-V3), presented in a form of Chinese familiar in Taiwan. Mean loneliness scores moved from the moderate range in pre-test to the mild range in immediate and three-month follow-up post-tests in the intervention group (from 42.24 points to 34.82 and 35). The control group of wait-listed volunteers had similar initial scores to the interventional group but showed no significant change at later measurement points (Chiang et al., 2009, p. 385). 

The nursing home study participants were of a narrower age range than would be found among the patients, families, and staff at a hospital. Yet the COVID-19 situation creates an enhanced parallel of isolation from the familiar wider world. The mutuality created in the small group, where participants are taught to listen attentively to others, and its effective amelioration of loneliness, may suggest forms that might be utilized by healthcare chaplains and programs we create.

## Exploration

### Loneliness, Evolutionary Psychology, and Social Neuroscience as Interpretive Resources in Healthcare Chaplaincy in the COVID-19 Context

The evolutionary psychology of Dunbar and collaborators and the social neuroscience of J. Cacioppo and collaborators suggest an interpretive and action shaping resource for healthcare chaplaincy in the context of COVID-19 and, perhaps, into the post-pandemic future.

Using this resource, it is possible to make plausible sense of my phone visit with “Lucia” described above. In COVID-19 quarantine she was lonely, missing mutual intimate, relational, and communal connection with others. In her customary life, Lucia may have intimate friends at her church. Perhaps close family members are fellow Catholics. In her religious life, Lucia likely has many relational connections, friends with whom she may chat and catch up after Mass, friends with whom she may carry out congregational or public service projects. Being Catholic and having the communal relationships that come with it may well be central to Lucia’s identity.Being quarantined and unable to attend Mass in person and so unable to connect with parish friends may be a relational and intimate loss.

If so, my call may have provided linkage with these customary connections with their endorphins and laughter. Brief formal prayer and familiar Bible passages in my telephone ministry may be effective in an isolated room in connecting with the memories and hopes–and endorphins–of public worship. Giving patients an opportunity to pray with the chaplain, to bless those who care for them, and to pray for others offers a way to mutuality even when in the grip of COVID-19.

Following the logic of the Dunbarand J. Cacioppo research, underutilization of programs for staff to express and work through frustrations, fears, or concerns makes sense. These are unidirectional opportunities. For the apt need, nothing could be better. But these opportunities are not mutual in design. They cannot meet our human need for connection the way interactions in which we both receive and give do.

### Loneliness, Evolutionary Psychology, and Social Neuroscience in the Creation of Translational Resources for Healthcare Chaplaincy in the COVID-19 Context

As I began to formulate these exploratory observations, I tested suggested insights in a visit with a patient. “Alicia” is in her late forties, Protestant, and African American. She was hospitalized with a psychotic diagnosis. Her medication was beginning to reach a therapeutic level, but she was still somewhat manic. Alicia asked for a Bible and a visit from a chaplain.

Reading her chart before my visit I saw that she had reported to staff that she had studied law and chaplaincy in the past. I was not surprised that, when we met, Alicia reported that she was also a chaplain. Although I had no external verification of the sense in which it might be literally accurate that she was a chaplain also, I took this as an opportunity to build mutuality with Alicia.

After an exchange during which I learned something of her current concerns and hopes, I led a prayer in the first-person plural. We prayed for our health and current needs, for the needs of patients around us and staff of the hospital, families at home, the needs of the world. Next, Alicia prayed for me, my needs, and my family. She continued with a joyous general praise toward the Lord and his goodness and care. We concluded with our good wishes for the day.

Alicia was not wearing a face mask and I knew from her chart this had been a matter of concern and frustration in the unit. As we met each other we spoke about the fact that we could not shake hands because it was possible that some virus had settled on me as I walked the corridor to visit her and I might pass that along to her. As I was leaving, I heard Alicia behind me asking the desk nurse to help her find a face mask to wear.

It seems plausible that our time of mutuality had given Alicia a renewed sense of being in a web of mutual relationships, and interest in behaving as if she was in a community in the unit. She wanted to continue to feel that she was both giving and receiving.

Mutuality is inherent in relating with others based in shared orientation toward spirituality or religion, toward a life of purpose and meaning. Other relationships at the hospital are primarily for receiving care. In pre-pandemic conditions, patients, families, and other visitors keep themselves connected to their customary intimate, relational, and communal/identity relationships in ways that the isolations of the COVID-19 context have made difficult. Chaplains are uniquely placed to enable patients to express their concerns for and connections with others, articulating a life context in which patients are givers as well as receivers.

Perhaps staff are not using the support opportunities offered, framed in the classic healthcare chaplaincy format of supportive listening to the processing of intense emotion, as much as has been hoped, because these opportunities do not, cannot, well address the loneliness, the feeling of being socially disconnected that the COVID-19 context is causing. Perhaps what is needed during COVID-19 is enhanced access to and support for mutual relationships in the workplace.

Such needs on the part of patients, families, and staff are what the basic science of Dunbar’s evolutionary psychology and J. Cacioppo's social neuroscience lead us to expect.Exploring how Dunbar’s and J. Cacioppo’s basic science can be translated into effective loneliness care could be a useful addition to existing modes.

The COVID-19 context precludes in-person groups for CBT or mutual connection. But webinars, podcasts, hand-outs, pamphlets, and booklets offering explanations of why people are feeling so disoriented by our disconnections and what might be helpful at this time would not be hard to create and distribute. Voluntary online sharing of reminiscences could be tested for effectiveness without extensive effort or expense.

## Further Research

To move forward in such translation efforts, healthcare chaplains would need to ask how we can utilize the basic science of evolutionary psychology and social neuroscience in practical chaplaincy successes, incorporating this research into care of patients, families, and staff, and creating new programs to provide additional service. Second, we would need to ask how we can frame, carry out, and document such explorations in ways that enhance our translation literature and the externally accessible rigor of the discipline of healthcare chaplaincy most effectively. These are compelling concerns at any time. The extraordinary nature of the COVID-19 context gives added urgency.

## Note

1 Memorare: Remember, O most gracious Virgin Mary, that never was it known that anyone who fled to thy protection, implored thy help, or sought thy intercession was left unaided. Inspired with this confidence, we turn to thee, O Virgin of virgins, our Mother. To thee we come, before thee we stand, sinful and sorrowful. O Mother of the Word Incarnate, do not despise our petitions, but in thy mercy hear and answer us. Amen.
